# Dr. L. A. Moore

**Published:** 1963-10

**Authors:** 


### OBITUARY

Dr. L. A. Moore died on 9th May, 1963, in a Truro Hospital, aged 85.

Leonard Augustine Moore was educated at Bristol Grammar School and the Medical School and the Royal Infirmary, Bristol. He qualified by taking the London Conjoint Diploma in 1900 and obtained the Bristol M.B., Ch.B. in 1910 when the then University College was granted its own charter.

He was appointed the first Dental Anaesthetist to the Bristol Royal Infirmary in 1904, and later became Honorary Assistant Anaesthetist, and subsequently Honorary Anaesthetist, which post he held until his retirement in 1942.

He was a past president of the Bristol Med. Chi. Society and of the Bristol Playgoers' Club, and was a prominent Freemason.

He was a man of great charm and friendliness, and his constant cheerfulness and gay chuckle made him popular with all members of both the medical and nursing staff at the Royal Infirmary; and although he retired so long ago, there are still a few members on the staff who remember him with the greatest affection and respect.

